# A Phase II, single-arm study of nivolumab in patients with metastatic or unresectable urothelial cancer who have progressed following treatment with a platinum agent

**DOI:** 10.1186/2051-1426-3-S2-P174

**Published:** 2015-11-04

**Authors:** Padmanee Sharma, Marc-Oliver Grimm, Matthew D Galsky, Ari Baron, Sergio Bracarda, Arlene Siefker-Radtke, Alexandre Lambert, Alex Azrilevich, Margitta Retz

**Affiliations:** 1 MD Anderson Cancer Center, Houston, TX, USA; 2 Department of Urology, University Hospital of Jena, Jena, Germany; 3 Icahn School of Medicine at Mount Sinai, New York, NY, USA; 4 California Pacific Medical Center, San Francisco, CA, USA; 5 Medical Oncology Unit, Department of Oncology, Istituto Toscano Tumori (ITT), Arezzo, Italy; 6 Bristol-Myers Squibb, Princeton, NJ, USA; 7 Klinikum Rechts der Isar, Department of Urology, Technische Universitat Munchen,, Munich, Germany

## Background

Clinical trials in patients with advanced bladder cancer have reported response rates of up to approximately 30% and 60% with single-agent and multi-agent regimens, respectively, and minimal improvements in survival over best supportive care. Guidance on second-line treatment options is unclear and no global standard exists. Nivolumab, a fully human IgG4 programmed death-1 (PD-1) immune checkpoint inhibitor antibody, has clinical activity in multiple tumor types. Safety and activity will be evaluated in a Phase I/II, open-label study of nivolumab alone or in combination with ipilimumab in patients with advanced or metastatic solid tumors, including bladder cancer (NCT01928394). In a separate study and the focus of this abstract (NCT02387996), we will estimate the effect of nivolumab on tumor size and other safety and efficacy parameters in patients with unresectable or metastatic urothelial cancer progressing or recurring following platinum-based chemotherapy.

## Methods

This multinational study for advanced urothelial cancer patients, which started in March 2015, is expected to enroll as many as 250 patients and will be completed in October 2017. Eligible patients have metastatic or surgically unresectable urothelial carcinoma with measurable disease by imaging; disease progression or recurrence with ≥1 prior platinum-based regimen; and no liver metastases if >2 prior lines of chemotherapy were administered. Additionally, patients who had cystectomy for localized urothelial cancer along with disease recurrence or progression within 12 months of neo-adjuvant or adjuvant platinum-based treatment are eligible. Patients with active CNS metastases are excluded. Nivolumab monotherapy will be administered every 2 weeks until disease progression or unacceptable toxicity. The primary endpoint is objective response rate, and secondary endpoints include progression-free survival and overall survival. Further sub-analyses will be performed by programmed death-ligand 1 (PD-L1) tumor expression.

## Trial registration

ClinicalTrials.gov identifier NCT02387996.

